# Unfolded protein response in colorectal cancer

**DOI:** 10.1186/s13578-021-00538-z

**Published:** 2021-01-29

**Authors:** Jingjing Huang, Huayang Pan, Jinge Wang, Tong Wang, Xiaoyan Huo, Yong Ma, Zhaoyang Lu, Bei Sun, Hongchi Jiang

**Affiliations:** 1 Department of General Surgery, Key Laboratory of Hepatosplenic Surgery, Ministry of Education, The First Affiliated Hospital of Harbin Medical University, Harbin, Heilongjiang Province 150001 People’s Republic of China; 2 The Second Affiliated Hospital & College of Nursing, Harbin Medical University, Harbin, People’s Republic of China; 3 Pediatrics Department of The First Affiliated Hospital, Harbin Medical University, Harbin, People’s Republic of China

**Keywords:** Colorectal cancer, Inositol requiring kinase 1, Pancreatic ER eIF2α kinase, Activating transcription factor 6, Unfolded protein response

## Abstract

Colorectal cancer (CRC) is a gastrointestinal malignancy originating from either the colon or the rectum. A growing number of researches prove that the unfolded protein response (UPR) is closely related to the occurrence and progression of colorectal cancer. The UPR has three canonical endoplasmic reticulum (ER) transmembrane protein sensors: inositol requiring kinase 1 (IRE1), pancreatic ER eIF2α kinase (PERK), and activating transcription factor 6 (ATF6). Each of the three pathways is closely associated with CRC development. The three pathways are relatively independent as well as interrelated. Under ER stress, the activated UPR boosts the protein folding capacity to maximize cell adaptation and survival, whereas sustained or excessive ER triggers cell apoptosis conversely. The UPR involves different stages of CRC pathogenesis, promotes or hinders the progression of CRC, and will pave the way for novel therapeutic and diagnostic approaches. Meanwhile, the correlation between different signal branches in UPR and the switch between the adaptation and apoptosis pathways still need to be further investigated in the future.

## Introduction

Colorectal cancer (CRC) is one of the most common alimentary canal malignancies. According to the International Agency for Research on Cancer statistics in 2018, CRC ranks third in terms of incidence but second in terms of mortality. CRC is also the third most common type of malignancy in men, after lung and prostate cancers, and the second most frequent malignancy in women, after breast cancer. The global burden of CRC is expected to reach approximately 2.2 million new cases per year in 2030, thus exhibiting a further 20% increase [1]. The 5-year cumulative survival is 64–67, 89–90% in patients with localized cancer, 70–71% in those with regional metastasis, decreasing to 14–15% in distant metastasis [2].

The mechanisms of CRC occurrence and progression are not fully elucidated yet. A growing number of researches have demonstrated that the unfolded protein response (UPR) is closely involved in CRC development. The UPR provides a novel orientation for the diagnosis, therapies, and prevention of CRC.

## Introduction of UPR

The endoplasmic reticulum (ER) is an organelle in the eukaryotic cell responsible for the synthesis, folding, modification, and quality control of numerous secretory and membrane proteins. It also provides an appropriate environment for lipid, steroid, and cholesterol biosynthesis [3]. The processes of protein folding can be disturbed by environmental changes in ER homeostasis (ER stress), such as Ca^2+^ depletion, oxidative stress, hypoxia, energy deprivation, metabolic stimulation, altered glycosylation, activation of inflammation, as well as increases in protein synthesis or the expression of unfolded/misfolded proteins or the unassembled protein subunits. Only correctly folded proteins can be transferred to the Golgi apparatus while those unfolded/misfolded ones are transmitted to ER-associated degradation (ERAD) [4, 5]. ERAD is activated to alleviate unfolded protein accumulation, enhance protein folding capacity in ER, and increase ER-related chaperones' expression to stabilize protein folding, including glucose-regulated protein 78 (GRP78; also known as BIP and HSPA5), glucose-regulated protein 94 (GRP 94), calnexin.

**Fig.1:**
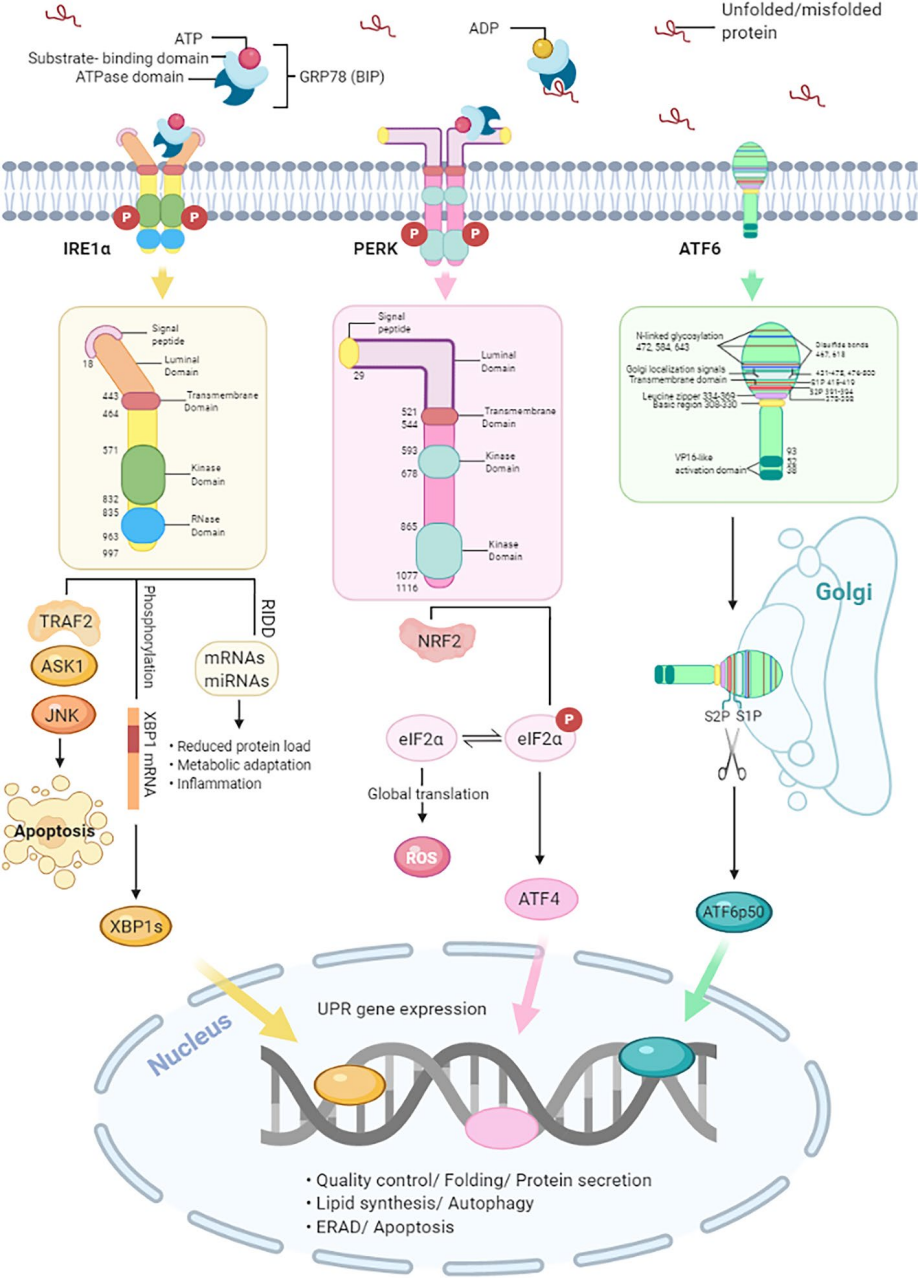
Schematic representation of the unfolded protein response (UPR) signaling pathways

In mammals, the UPR signal pathway is mediated by three ER transmembrane protein sensors: inositol requiring kinase 1 (IRE1), pancreatic ER eIF2α kinase (PERK), and activating transcription factor 6 (ATF6) [5]. In response to ER stress in the early phase, the UPR is initiated to orchestrate proper protein folding and degradation of unfolded/misfolded protein as an adaptive pathway for survival. Under ER stress, the chaperone GRP78, which initially binds to three ER transducer sensors' luminal domain, dissociates from them. The activated UPR transduced by the three pathways potentiate adaptation and survival capacity, while the overwhelmed UPR leads to apoptosis under ER stress (Fig. 1).

The UPR is activated by the accumulation of unfolded/misfolded proteins in the endoplasmic reticulum (ER) lumen when glucose-regulated protein 78 (GRP78; also known as BIP and HSPA5) dissociates from the three ER stress sensors: inositol requiring kinase 1 (IRE1), pancreatic ER eIF2α kinase (PERK), and activating transcription factor 6 (ATF6). Firstly, the oligomerization of IRE1α leads to its RNase activation, which promotes the production of the transcription factor X-box binding protein 1 splicing (XBP1s) translocated to the nucleus and potentiated the expression of genes. Besides, IRE1α activation triggers the degradation of ER-associated mRNAs by regulating IRE1-dependent decay of mRNAs (RIDD), reducing protein load and promoting metabolic adaptation. IRE1α can also facilitate c-Jun N-terminal kinase (JNK) signaling, resulting in the induction of apoptosis. Secondly, UPR signaling can cause transcriptional blockade via the PERK mediated phosphorylation of eukaryotic translation initiation factor 2 (eIF2α). Though global protein synthesis is inhibited, activating transcription factor 4 (ATF4) is translated, up-regulating genes involved in autophagy and amino acid metabolism. Signaling through the UPR aims to restore ER homeostasis by blocking the further build-up of unfolded proteins, enhancing the folding capacity, and initiating misfolded proteins' degradation. However, apoptotic signaling such as C/EBP homologous protein (CHOP) will be induced, and programmed cell death will increase with persistent ER stress. Thirdly, activated activating transcription factor 6 (ATF6α) is cleaved in the Golgi by the site1 proteases (S1P) and site2 proteases (S2P) to produce a transcription factor, which can be translocated to the nucleus and induce related gene expression.

### IRE1 signaling pathway

IRE1 is the most conserved branch of the UPR, a type I transmembrane protein with both a serine/threonine kinase domain and an endoribonuclease (RNase) domain in its cytosolic portion. IRE1α is expressed ubiquitously in mammals, whereas IRE1β expression is mostly restricted to the intestinal and other mucosal surfaces. Activation of IRE1 starts from the dissociation from GRP78, the luminal domain of IRE1α forms homodimers in the plane of the ER membrane. However, it may be assembled with IRE1β to form other hypoactivity oligomeric complexes [6], the kinase domains for trans-autophosphorylation to boost the kinase and RNase activities. These activities initiate the removal of a 26-base intron from the mRNA encoding X-box-binding protein 1 (XBP1), resulting in a translational frameshift, which generates a 41 kDa CREB/ATF (cAMP-response element-binding protein/Activating transcription factor) basic leucine zipper (bZiP)-containing transcription factor. X-box binding protein1splicing (XBP1s) spliced from XBP1 launches a transcriptional program. This program potentiates the production of chaperones alone or with other transcription factors, proteins involved in ER biogenesis, phospholipid synthesis required for ER expansion under ER stress, ERAD, and secretion. For example, ER degradation-enhancing alpha mannosidase-like 1 (EDEM), ER-localized DnaJ 4 (Erdj4) and protein disulfide isomerase (PDI) [7]. Therefore, IRE1α-XBP1s signaling is one of the major pathways for enhancing the ER's folding capacity and dealing with ER stress [8]. Also, unfolded proteins can bind directly to the luminal domains of IRE1α, facilitating the assembly of highly ordered IRE1α clusters, which may orient the cytosolic region of the dimer to create a ribonuclease site and generate an mRNA docking region [9, 10].

Besides selective cleavage of the XBP1 mRNA, IRE1 degrades a subset of ER-localized mRNAs in drosophila and mammalian cells in a stress-dependent manner termed regulated IRE1-dependent decay of mRNAs (RIDD). Thereby protein synthesis is attenuated to alleviate ER stress. Phosphorylation and activation of IRE1α also result in the recruitment of the adaptor protein tumor necrosis factor receptor (TNFR)-associated factor 2 (TRAF2) and apoptosis signal-regulating kinase 1 (ASK1) to the cytoplasmic leaflet of the ER membrane, and then, they elicit a cascade of phosphorylation events that target c-Jun N-terminal kinase (JNK) [11, 12]. Subsequently, apoptosis is promoted (Fig. 1).

### PERK signaling pathway

PERK is a type I transmembrane protein with a cytosolic serine/ threonine kinase domain, which is the most immediate sensor for ER stress. Upon ER stress, the chaperone GRP78 releases from PERK, which then undergoes oligomerization and trans-auto phosphorylation [5, 13]. The activation mechanism of PERK is quite similar to that of IRE1α. Activated PERK phosphorylates Ser51 of the subunit of eukaryotic translation initiation factor 2 (eIF2α), which in turn attenuates translation initiation. The decrease in global translation quickly reduces the amount of newly synthesized proteins entering the ER, thereby decreasing the ER protein-folding load. Despite a stop in translation, a few selected mRNAs with short upstream open reading frames (uORF) in the 5′-UTR escape from translational inhibition [14]. For example, it activates transcription factor 4 (ATF4), regulating the expression of genes involved in redox balance, amino acid metabolism, protein folding, autophagy, and cell survival [15, 16]. C/EBP homologous protein (CHOP/GADD153) is one of the ATF4 target genes, encoding a transcription factor involved in apoptosis regulation [17]. PERK signaling is fine-tuned by the CHOP target gene growth arrest and DNA damage-inducible 34 (GADD34), which associates with the phosphatase protein phosphatase 1 (PP1), then dephosphorylation of eIF2α is enhanced, thereby alleviating translational inhibition [16, 17]. Therefore, PERK signaling is central in the switch between the adaptive response phase and chronic ER stress leading to apoptosis. In addition, CHOP promotes oxidative protein folding in the ER through the induction of ER oxidoreductin-1 alpha (ERO1α) expression. However, the accompanying increase in disulfide bond formation generates reactive oxygen species (ROS) [18, 19], Which might explicate that under conditions of chronic ER stress, the CHOP-mediated increase in protein flux into the ER through GADD34 [20], and the subsequent increase in ROS formation can, in turn, lead to enhanced stress exacerbating apoptosis [19, 21]. The UPR response can orchestrate itself and correct automatically and cope with the ROS's effect by activating an antioxidant response. PERK activates the transcription factors ATF4 and nuclear factor E2 related factor 2 (NRF2), increasing genes involved in antioxidation [17]. NRF2 is mainly held in the cytoplasm. Phosphorylation of NRF2 by PERK triggers its dissociation and nuclear import [22]. PERK-eIF2α signaling also activates nuclear transcription factor-κB (NF-κB) through translational repression of the inhibitor of kappa B (IκB), leading to regulation of apoptosis [23]. The co-chaperone P58IPK targets both XBP1s and ATF6α, and it inhibits PERK signaling by interacting with the kinase domain of PERK and impairing eIF2α phosphorylation [24–26] (Fig. 1).

In humans, three other eIF2α kinases can phosphorylate eIF2α and regulate translation: general control nonderepressible-2 (GCN2) activated by nutrient deprivation [27], heme-regulated initiation factor 2 alpha kinase (HRI) induced by heme deficiency and oxidative stress [28–30], and protein kinase interferon-inducible double-stranded RNA dependent (PKR) which is elicited by viral infection [31, 32]. They all converge on the same residue's phosphorylation in eIF2α and are collectively referred to as the integrated stress response (ISR) [17].

### ATF6 signaling pathway

Effector ATF6 is the causal role of the UPR in tumor biology. Little is known about it, although its downstream target gene GRP78 is frequently found to overexpress. ATF6 is a type II ER transmembrane protein with a CREB/ATF bZip transcription factor domain at the amino terminus. Upon accumulation of ER stress, ATF6 dissociates from GRP78 for trafficking to the Golgi apparatus wherein it is sequentially cleaved by site 1 and site 2 proteases at the transmembrane site, yielding a cytosolic fragment known as ATF6 p50, which migrates to the nucleus to activate gene expression [5, 33]. The ATF6 luminal domain also contains intra- and intermolecular disulfide bonds that may monitor the ER environment as redox sensors [34]. Target genes of ATF6α include chaperones GRP78, GRP94, ERAD components, the UPR genes XBP1, protein kinase inhibitor of 58 kDa (P58IPK/DNAJC3), and CHOP [35–38]. ATF6α can also heterodimerize with XBP1s to regulate transcription from UPR elements in target genes [37]. Similar to ATF6α, ATF6β is cleaved and transferred to the nucleus upon ER stress. However, ATF6β is a poor transcriptional activator and appears to repress ATF6α-mediated induction of UPR targets, suggesting that ATF6β may serve as an endogenous inhibitor of ATF6α [39] (Fig. 1).

### The interreaction between UPR pathways

**Fig.2:**
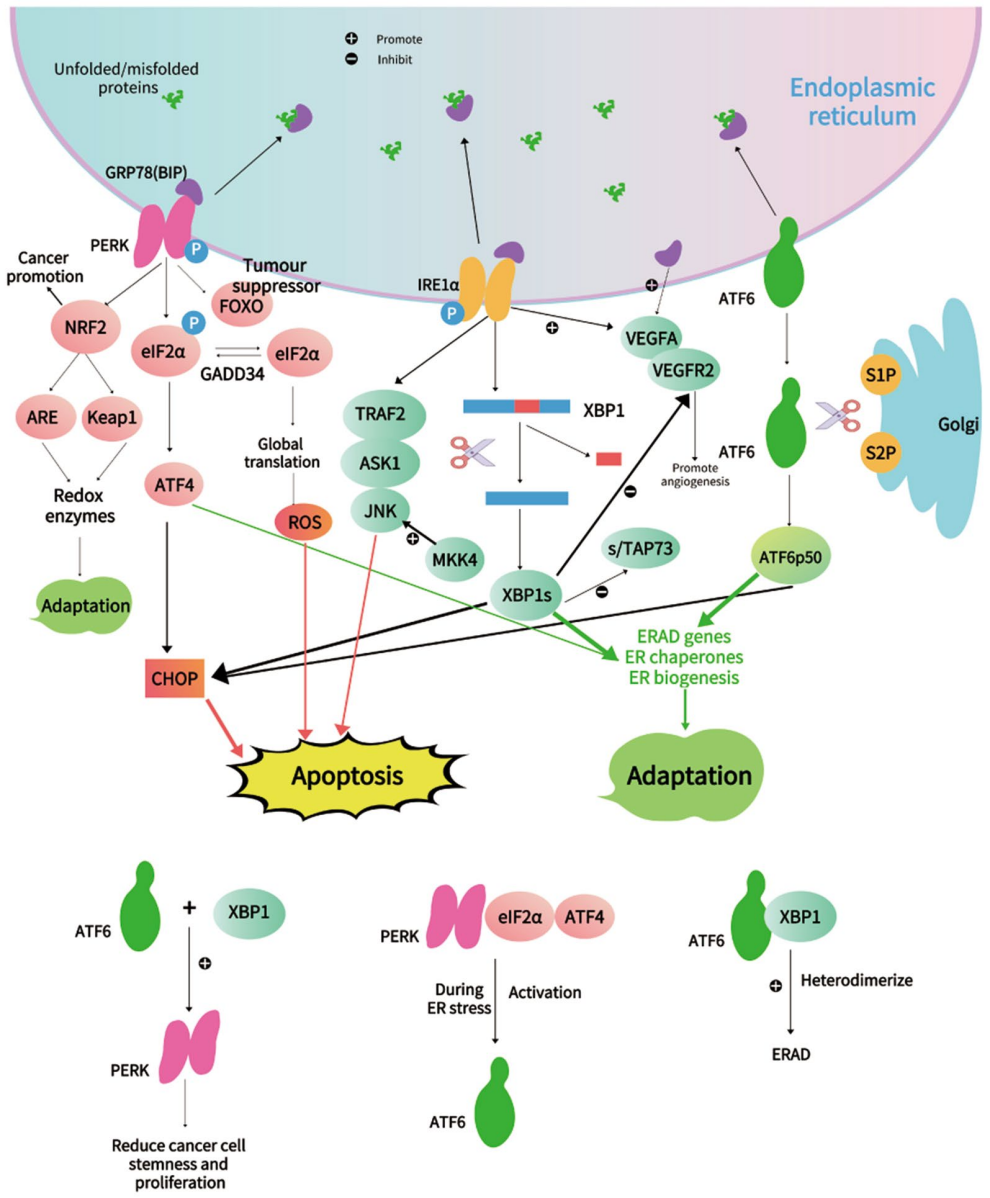
Other significant pathways and regulatory factors in UPR

Recently, a study has confirmed that the PERK/ eIF2α/ATF4 pathway is central for the activation of ATF6 during ER stress [40]. Also, it has shown that ATF6α heterodimerizes with XBP1 for the induction of ERAD. ATF6 and XBP1 heterodimerize in vivo when expressed in ER-stressed cells, and ATF6-XBP1 heterodimer is bound to the UPR element in vitro. Furthermore, ATF6-XBP1 heterodimer possesses eightfold higher affinity to the UPR element than XBP1 homodimer [37] (Fig. 2).

Studies uncover novel crosstalk between activated XBP1 and ATF6 and PERK-eIF2α. They generated colorectal cancer cells (LS174T) that harbor doxycycline-inducible expression of the active forms of either XBP1s or ATF61-373. Activation of either XBP1 or ATF6 resulted in reduced cellular proliferation and declined expression of intestinal epithelial stemness markers. Furthermore, XBP1 and ATF6 activation overwhelmingly attenuates global protein synthesis and lowers the threshold for UPR activation. XBP1 mediated loss of stemness and proliferation results from cross-activation of PERK-eIF2α signaling and could be rescued by constitutive expression of eIF2α phosphatase GADD34. Therefore, the synergistically enforced activation of XBP1 and ATF6 results in the reduction of stemness and proliferation. In light of these findings, it may provide a novel strategy to target PERK-eIF2α for the treatment or prevention of intestinal malignancies [41] (Fig. 2).

## ER stress-mediated activation of the UPR act as a double-edged sword in cancer

The UPR is a double-edged sword in cancer development. During ER stress, cells either survive by inducing adaptation mechanisms or suicide by apoptosis. ER stress and UPR activation play crucial roles in the various stages of cancer [17, 42, 43] (Fig. 2).

### The UPR promotes cancer-cell adaptation

The UPR signaling attenuates mRNA translation to ameliorate overloaded unfolded/misfolded proteins. Besides, the expression of chaperones repairing unfolded/misfolded proteins is increased [44]. During ER stress, cancer cells tend to modify their ER-resident proteins and chaperones to increase cell viability. When using UPR inhibitors, such as 4-PBA (4-Phenylbutyric acid) or TUD-CA (Tauroursodeoxycholic acid), cancer progression and metastasis are significantly decelerated [45]. Activated IRE1 can promote the expression of XBP1 and induce ERAD and further facilitate cell survival [46]. PERK can also increase cancer cell viability during adversities, such as hypoxia, ATP shortage, and nutrient deficiency [47].

In addition to the above, cancer cells can also protect themselves for survival through UPR-driven immunosuppression. During ER stress, cancer cells can actively modulate immunocytes function through transmissible ER stress. It has been identified that activated ER stress in cancer cells can promote UPR genes and proinflammatory cytokines in responder macrophages [48]. This study has also verified that the cell-extrinsic effects of tumor ER stress imprint myeloid DCs and impair CD8 + T cell priming [49]. Further evidence has been discovered to confirm the modulation of immunocytes through ER stress that ER stress in tumor-bearing mice accelerated cancer progression and the immunosuppressive capacity of myeloid-derived suppressor cells (MDSCs) [45].

Besides, UPR also plays a crucial part in dormancy associated with cancer cell survival and chemoresistance [47, 50, 51].

In the PERK signaling pathway: CHOP is a vital mediator of stress-induced apoptosis. The PERK-eIF2α-ATF4-CHOP is a critical pathway of CHOP that caused apoptosis. Besides, ATF6 also has an essential contribution to CHOP production at early time points, and XBP1 regulates CHOP to a minimal extent. Under sustained chronic ER stress, the CHOP-mediated elevation in protein flows into the ER through growth arrest and DNA damage-inducible 34 (GADD34), and the subsequent increase in reactive oxygen species (ROS) formation can, in turn, lead to aggravative ER stress and thus cell death. The downstream transcription factor of PERK, namely nuclear factor E2 related factor 2 (NRF2), involves cellular adaptation and cancer promotion. The NRF2-Kelch-like ECH-Associating protein 1 (Keap1), as well as the NRF2-antioxidant response element (ARE), can counteract the harmful effects of reactive oxidants in cancer cells and restore redox balance to promote cancer adaptation. Forkhead box O (FOXO) is a non-canonical pathway of PERK that serves as a tumor suppressor, exerts its function by promoting cell cycle arrest and apoptosis, preventing the accumulation of damages induced by genotoxic agents and oxidative stress. In the IRE1 signaling pathway, MKK4, a MAP3K upstream of JNK, can activate JNK to suppress metastasis. p73, as a member of the tumor suppressor p53 family, encodes protein TAp73, XBP1-s/TAp73 axis attenuates colorectal cancer cell proliferation. The activation of the ER stress sensor IRE1, identified as a common determinant linking hypoxia and hypoglycemia dependent responses to the up-regulation of VEGF-A. The combination of VEGFA and VEGFR2, a transmembrane tyrosine kinase receptor involves in the regulation of angiogenesis, XBP-1 remarkably increases the level of VEGFR2.

The interreaction between UPR pathways: serving as UPR effector proteins, ATF6 and XBP1, mitigate colorectal cancer cell proliferation and stemness by activating PERK signaling. Besides, the PERK/eIF2α/ATF4 pathway is central for activation of ATF6during ER stress. Additionally, ATF6α heterodimerizes with XBP1 to elicit the ERAD machinery. ATF6-XBP1 heterodimer exhibits eightfold higher affinity to the UPR element than XBP1 homodimer.

### UPR leads to cell death through multiple mechanisms

ATF6, ATF4, and XBP1s act in parallel to activate CHOP and then induce apoptosis. The IRE1-mediated JNK pathway could elicit both apoptotic and non-apoptotic cell death [52]. Besides, the generation of ROS can also result in apoptosis.

CHOP is a canonical pro-apoptotic factor under ER stress. CHOP (a 29 kDa bZIP transcription factor) is a vital mediator of stress-induced apoptosis. It triggers activation of several pro-apoptosis factors, including building information modeling (Bim), a pro-apoptotic member of B-cell lymphoma 2 (BCL-2) family, death receptor 5 (DR5), telomere repeat binding factor 3 (TRB3), and abolishes the expression of the anti-apoptotic protein BCL-2 [5, 53, 54].

The promoter of CHOP contains the binding sites for several major trans-activators of the UPR, including ATF4, ATF6, and XBP1s. The PERK-eIF2α-ATF4-CHOP signaling is identified as a vital pathway of CHOP caused apoptosis. Additionally, ATF6 also has been uncovered to potentiate CHOP production at early time points. It has been well verified that the ATF6 branch shapes the early dynamics of CHOP production, whereas ATF4 dominates the CHOP production at late time points. Furthermore, CHOP can also be modulated by XBP1 to a minimal extent [36, 55–57].

Moreover, as mentioned above, CHOP leads to oxidative stress through inducing ERO1α, ERO1α transfers electrons from protein disulfide isomerase to O_2_ for H_2_O_2_ production. ERO1α also promotes the release of Ca^2+^ from the ER through the inositol 1, 4, 5-trisphosphate receptor. Since Ca^2+^ is indispensable for ER chaperone function and protein folding, depletion of ER Ca^2+^ further impairs protein folding capacity. Ca^2+^ released from the ER is loaded into mitochondria, leading to oxidative stress and pro-apoptotic signaling [5, 58].

Collectively, the CHOP deficiency is not conducive to ER-stress-induced apoptosis. Therefore, CHOP could be considered a promising target for cancer therapy.

### UPR plays inverse roles in cancer metastasis

PERK is the most dominant branch of UPR to accelerate metastasis, as it can promote angiogenesis through increasing expression of vascular endothelial growth factor (VEGF), which serves as the most substantial angiogenesis stimulating factor and maintains endothelial cell survival, including VEGFA, VEGFB, VEGFC, VEGFD, VEGFF and placental growth factor (PIGF) [59]. Epithelial-to-mesenchymal transition (EMT) is a cell trans-differentiation program involved in migration and invasion [60–63]. Cancer cells that have undergone the EMT are prone to employ the PERK-ATF4 branch of UPR for metastasis [64].

Whereas, mitogen-activated protein kinase kinase (MKK4), a MAP3K upstream of JNK, has been found to hinder metastasis in prostate cancer by activation of JNK [65, 66]. Transfection of MKK4 in prostate cancer cells, which lack MKK4 expression, significantly recovers metastasis suppression but not growth inhibition of the primary tumor [67].

In summary, UPR is closely related to cancer progression, and there is increasing evidence that the function of UPR in cancer exhibits different sides. (Fig. 2).

Berberine (BBR) inhibits proliferation and migration of cancer cells by down-regulating the expression of GRP78. The anti-tumor activity of mung bean trypsin inhibitor (mTI) is attributed to the targeted suppression of GRP78. Paclitaxel triggers apoptosis by interacting with the mitogen-activated extracellular signal-regulated kinase/extracellular regulated protein kinases (MEK/ERK) pathway, closely associated with the down-regulation of GRP78. (-)-Epigallocatechin gallate (EGCG) binds to GRP78, suppresses tumor growth and enhances the sensitivity of colorectal cancer to 5-fluorouracil (5-FU). Studies indicate that up-regulation of CD24 might be a feasible mechanism of resistance in colorectal cancer CRC, improving CRC cells' sensitivity to oxaliplatin (L-OHP)-induced cytotoxicity by GRP78 suppression, is closely correlated with down-regulation of CD24. Fucoidan can inhibit the viability of cancer cells by interfering with the IRE1-XBP1 pathway and up-regulating elF2α-CHOP expression. 2-(3,4-dihydroxy phenyl) ethanol (DPE) elicits growth arrest and apoptosis in human colon carcinoma cells through regulation of IRE1-JNK signaling and activation of PERK-eIF2α-CHOP signaling. The PERK-ATF4-CHOP UPR branch is proven to be activated by small-molecule inhibitor 42,215. Brefeldin A (BFA) alleviates the progression of colorectal cancer during the tumorigenesis and metastasis stages via CHOP stimulation. Resveratrol can be used to treat CRC, accompanied by activation of eIF2α, CHOP cleavage of caspase-4 is also up-regulated in CRC cells. Piperine generates ROS, CHOP, JNK, cytochrome c, leading to cell death. Curcumin enhances the expression of CHOP and JNK to aggravate apoptosis. Combined Carfilzomib (CFZ) and the aggresome inhibitor ACY-1215 (Histone deacetylase 6-selective inhibitor) treatment resulted in significantly increased expression of eIF2α/ATF4/ CHOP and IRE1α/JNK.

## Three UPR pathways and chaperones in CRC

### Mechanism of GRP78 in cancer

GRP78, also known as the immunoglobulin heavy chain binding protein (BIP), belongs to the heat shock protein 70 (HSP70) family. GRP78 has a signal peptide sequence that targets it to the ER as a molecular chaperone [5, 68, 69], involves proper protein folding and assembly, proteasome degradation of unfolded/misfolded protein, ER Ca^2+^ binding, and orchestrates the activation of transmembrane ER stress sensors. GRP78 might bind misfolded proteins through the substrate-binding domain (SBD), which transduces a signal to the ATPase domain to release the repressive interaction over IRE1α and PERK. The expression of GRP78 elevates in various solid tumor types, including colorectal cancer, and recent studies have reinforced that GRP78 exhibits dual characteristics in cancers. On the one hand, GRP78 restrains early tumor development through numerous suppressive mechanisms such as dormancy induction [68, 70]. On the other hand, at advanced stages of cancer progression, when cancer cells are exposed to excessive ER stress, GRP78 has been discovered to promote cancer progression through its pro-survival [71] and pro-metastatic functions [72]. GRP78 on the cancer cell surface transmits cell membrane signaling pathways, thereby regulates proliferation, apoptosis, and tumor immunity [73, 74]. Besides, GRP78 also plays a crucial role in tumor angiogenesis, attributed to induced VEGF accommodation [75, 76].

The expression of GRP78 has been well characterized in CRC tissues by immunohistochemistry. In comparison with normal colon tissues, the representative results illustrate that the majority of histological sections of CRC tissues displayed enhanced expression of GRP78, and the immunoreactivity score (IRS) was significantly higher in metastatic and poorly differentiated tissue samples [77]. These results collectively indicate that GRP78 is constitutively increased in cultured CRC cell lines and CRC tissues and plays a profound role in regulating CRC cells' sensitivity to apoptosis induced by chemotherapy [78]. It is proposed that the pre-evaluation of the expression of GRP78 can serve as a useful biomarker for the response of CRC patients to DNA-targeting agents. Meanwhile, a baseline for personalized treatment of CRC patients can be established [79].

**Fig.3:**
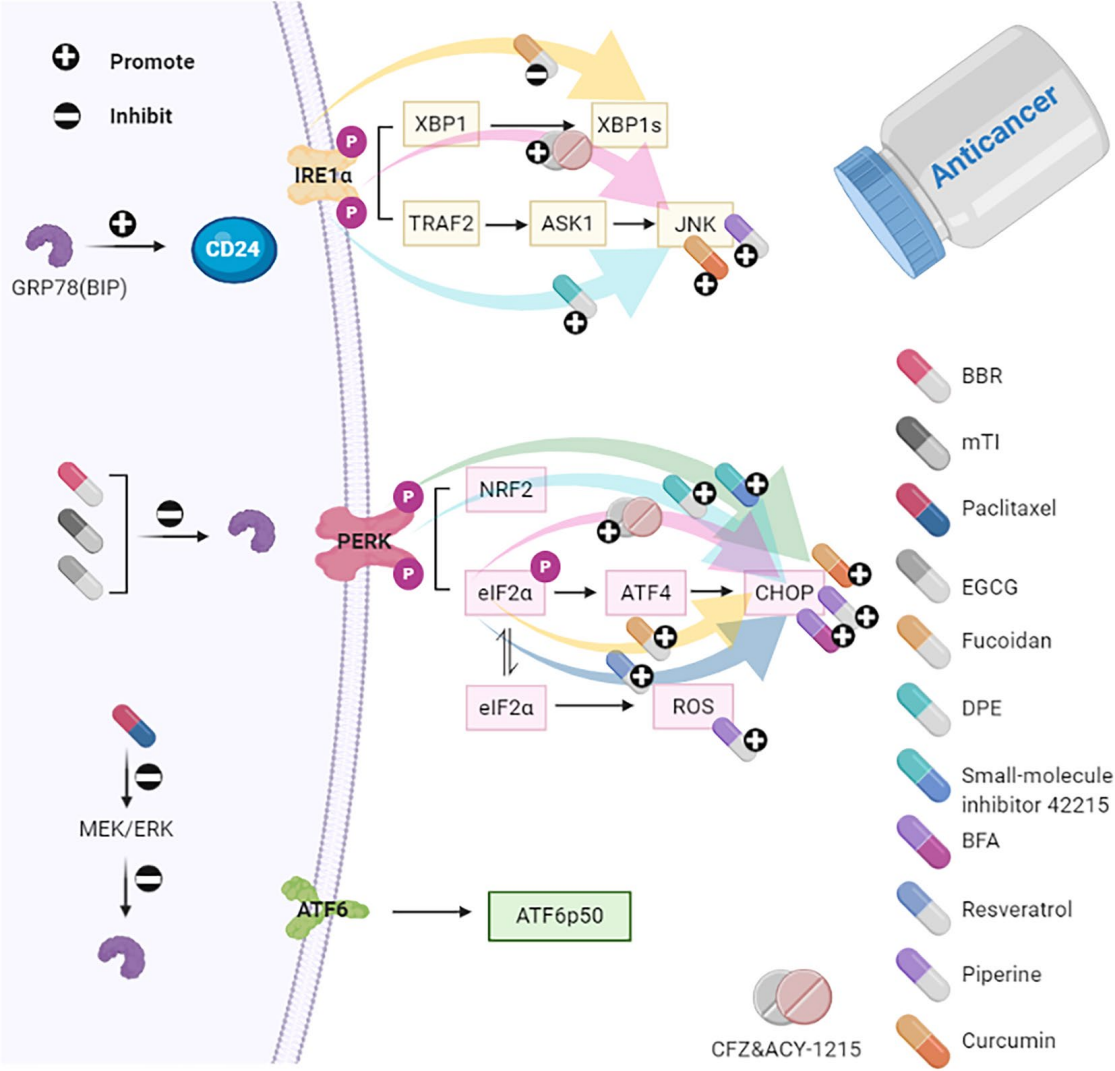
Schematic representation of how the drugs affect UPR in colorectal cancer (CRC)

1. Many drugs aim to inhibit GRP78 for the treatment of CRC
  Berberine (BBR) is an isoquinoline alkaloid isolated from berberis and coptis. It has been reported that BBR possesses anti-tumor effects in various human cancer cells. A previous study has demonstrated that BBR inhibited proliferation and migration of SW480 cells by attenuating the expression of GRP78 [80]. Trypsin inhibitors generally distribute in plants and animals, and some types of trypsin inhibitors display pronounced anticancer effects. For instance, the 33 residues, derived from the lysine active fragments of mung bean trypsin inhibitor (mTI), exert a potent activity against trypsin, the growth of colon cancer cells was dramatically restricted in response to mTI.
  Furthermore, a fusion protein containing the GRP78 binding peptide WIFPWIQL and mTI was constructed, and its specific anti-tumor effects were evaluated both in vitro and in vivo. The study has confirmed that the targeted anti-tumor activities are attributed to its interaction with cell surface GRP78 and subsequent cellular internalization [81]. Similarly, correlative data suggested that colorectal cancer cells' sensitization to paclitaxel-induced apoptosis inhibits the mitogen-activated extracellular signal-regulated kinase/extracellular regulated protein kinases (MEK/ERK) pathway, which was closely associated with the reduction of GRP78. Thus, combining compounds with an inhibitory capacity to GRP78 might be a novel approach for improving paclitaxel's effectiveness in treating colorectal cancer [82] (Fig. 3).
2. GRP78 attenuates chemotherapy sensitivity of colorectal cancer cells

A study indicates that the levels of GRP78 were negative in correlation with the sensitivity of CRC cells to chemotherapy-induced apoptosis. Knockdown of GRP78 by siRNA significantly restored the sensitivity of CRC cells to chemotherapeutic agents [78]. GRP78 detection provides an optional biomarker, determines the response to fluoropyrimidine-based adjuvant chemotherapy. GRP78 has been identified to predict benefit from 5-fluorouracil (5-FU)-based adjuvant chemotherapy and evaluate response to 5-FU in CRC cells [79].

(-)-Epigallocatechin gallate (EGCG) is active catechin isolated from green tea, which suppresses tumor growth and enhances drug sensitivity in various cancers, available data show that EGCG serves as a novel chemo-sensitizer, and the GRP78/NF-κB/miR-155-5p/MDR1 pathway plays a vital role in EGCG enhancing the sensitivity of colorectal cancer to 5-FU [83]. Besides, studies indicate that CD24 antagonizes oxaliplatin (L-OHP)-induced cytotoxicity. High expression of CD24 may be a potential mechanism mediated resistance in CRC cells. GRP78 was found to promote CD24 expression. Sensitization of CRC cells to L-OHP-induced cytotoxicity by inhibition of GRP78 was closely associated with low expression of CD24. Therefore, GRP78 repression may synergistically enhance the effectiveness of L-OHP in the treatment of CRC [84, 85].

(3) GRP78 is a biomarker for prognosis and early diagnosis of CRC.

The expression of GRP78 is an independent marker of survival in CRC [79]. Furthermore, the GRP78 rs391957 polymorphism promoter can also predict clinical outcomes in locally advanced CRC patients [86]. A recent report indicated a significant difference in the serum levels of anti-GRP78 antibodies between healthy subjects and polyp-bearing patients. Comparing the seven years of follow-up for healthy subjects and polyp patients revealed no differences between patients with higher or lower values of anti-GRP78 antibodies, which suggests that the presence of anti-GRP78 antibodies in patients' serum with CRC is a potential biomarker for early diagnosis but not prognosis [87].

### IRE1 pathway

IRE1 has at least three established outputs: XBP1 mRNA splicing, RIDD of other mRNAs, and direct interactions with downstream mediators [88]. Increased XBP1 splicing has been demonstrated in numerous cancers and is associated with more malignant phenotypes and poor survival [89–91]. Activated IRE1 recruits the adaptor protein TRAF2 to the ER membrane, which has been reported to further interact with JNK, resulting in caspase-12 activation and subsequently apoptosis in a mouse model [92]. These results suggest that IRE1-mediated apoptosis may be a strategy for anticancer therapy (Table 1).

1. IRE1 is essential in CRC angiogenesis
  Angiogenesis represents a crucial step in tumor development. Solid tumors initially occur in the absence of vascularization and then are subjected to various growth restrictions due to ischemia, accompanied by hypoxia and glucose deprivation. Furthermore, the UPR will be induced in tumors concomitant with angiogenesis. In an experiment, it has been proposed that the activation of IRE1 is a common determinant linking hypoxia and hypoglycemia dependent responses to the overexpression of VEGF-A [93]. Cancer cells expressing a dominant-negative IRE1 and embryonic fibroblasts derived from IRE1α-null mouse were unable to boost the expression of VEGF-A under either oxygen or glucose deprivation. Therefore, these data suggest that IRE1-dependent signaling pathways play an indispensable part in response to ischemia, and IRE1 is identified as a potential therapeutic target to control both the angiogenic switch and cancer development [94]. Surprisingly, hyperactivation of IRE1α in XBP1-deficient epitheliums drives the regenerative intestinal stem cell (ISC) expansion upon pathological ER stress but not involves in homeostatic ISC regulation [95].
2. XBP1 serves as a biomarker in CRC invasion and metastasis
  The XBP1 IRS expression is positively correlated with tumor invasiveness. Over-expression of XBP1 accelerates cancer cell invasion, suppressed by knockout of XBP1 using small interfering RNA (siRNA). Inhibition of XBP1 expression decreases levels of VEGF receptor-2 (VEGF-R2), a transmembrane tyrosine kinase receptor. VEGFA and VEGFR2 are synergistically involved in the regulation of angiogenesis. Thus, XBP1 might be identified as a novel predictive biomarker of CRC invasion and metastasis [96].
3. The XBP1s/TAp73 axis

**Table 1:** The UPR regulators in CRC and their functions, targeting drugs, biomarkers and literatures

Regulators	Functions	Targeting drugs	Biomarkers	Literatures
ARE	•Counteract the harmful effects of reactive oxidants •Restore redox balance			[99, 100]
ASK1	Regulate apoptosis signal			[11, 12]
ATF4	Regulating the expression of genes involved in redox balance, amino acid metabolism, protein folding, autophagy and cell survival	CFZ&ACY-1215		[15, 16]
ATF6	Induce ER biogenesis, chaperone up-regulation and unfolded/misfolded proteins degradation		A biomarker to distinguish LGD from inflammatory regenerative epithelium in UC patients	[38, 115, 119]
CHOP	Induce apoptosis	•Fucoidan •Small-molecule inhibitor 42,215 •2-(3,4-dihydroxyphenyl) ethanol (DPE) •Brefeldin A (BFA) •Resveratrol •Piperine •Curcumin •CFZ&ACY-1215		[17, 120–126]
EIF2ɑ	Translation initiation	•Fucoidan •Small-molecule inhibitor 42,215 •2-(3,4-dihydroxyphenyl) ethanol (DPE) •Resveratrol •CFZ&ACY-1215		[120–122, 124]
FOXO	Suppress tumour by promoting cell cycle arrest and apoptosis and preventing the accumulation of damages induced by genotoxic agents and oxidative stress			[102–104]
GRP78	•Involves in proper protein folding and proteasome degradation of unfolded/misfolded protein •ER Ca2 + binding •Orchestrates the activation of transmembrane ER stress sensors	•Berberine (BBR) •Mung bean trypsin inhibitor (mTI) •Paclitaxel (-)-•Epigallocatechin gallate (EGCG)	•A biomarker for prognosis and early diagnosis of CRC •A biomarker for response of CRC patients to DNA-targeting agents •A biomarker determines response to fluoropyrimidine-based adjuvant chemotherapy	[68, 79–83, 86, 87]
IRE1ɑ	•Induce ERAD to facilitate cell survival •Interact with JNK, resulting in cell apoptosis •Promote angiogenesis	•Fucoidan •2-(3,4-dihydroxyphenyl) ethanol (DPE) •CFZ&ACY-1215		[46, 92, 93, 120, 121]
JNK	Induce apoptosis	•2-(3,4-dihydroxyphenyl) ethanol (DPE) •Piperine •Curcumin •CFZ&ACY-1215		[11, 121, 125, 126]
KEAP1	•Counteract the harmful effects of reactive oxidants •Restore redox balance			[99, 100]
MKK4	Hinder metastasis			[65–67]
NRF2	Increasing genes involved in antioxidation			[17]
PERK	Is central in the switch between the adaptation and apoptosis	•Small-molecule inhibitor 42,215 •2-(3,4-dihydroxyphenyl) ethanol (DPE)		[16, 17, 121, 122]
ROS	Increase cell death	Piperine		[19, 125]
s/TAP73	Promoting CRC cell proliferation and colony formation			[97]
TRAF2	Interact with tumor necrosis factor receptor and many other signaling molecules			[11, 12, 92]
VEGFA	Stimulate angiogenesis			[59, 96]
VEGFR2	Stimulate angiogenesis			[59, 96]
XBP1	Mediated stemness, proliferation and metastasis	Fucoidan	A biomarker in CRC invasion and metastasis	[35, 37, 41, 89, 95, 96, 120]

p73, together with p53 and p63, is a member of the tumor suppressor p53 family; the p73 gene encodes full-length protein isoform (TAp73), crucial for XBP1s-induced tumorigenesis. A study suggests the XBP1s/TAp73 axis’s critical role in promoting colorectal cancer cell proliferation and colony formation. These findings implicate the potential of targeting the XBP1s/TAp73 axis for CRC [97] (Fig. 2).

### PERK pathway

Two downstream transcription factors of PERK, namely ATF4 and NRF2, contribute to cellular adaptation and oncogenesis [98]. NRF2 regulates the inducible expression of antioxidant response element (ARE) containing genes. The NRF2-Kelch-like ECH-Associating protein 1 (NRF2-Keap1), as well as the NRF2-ARE, can counteract the harmful effects of reactive oxidants in mammalian cells and restore redox balance to promote cancer progression [99, 100] (Fig. 2).

1. The loss of forkhead box O (FOXO) promotes the progression of CRC
  FOXO is also a significant pathway of PERK. Accumulating evidence suggests that PERK can phosphorylate FOXO and facilitate its activity [101]. FOXO transcription factors family are known to acts in synergy with growth and survival factors under various stress events. They are recognized as tumor suppressors in the light of their functions in promoting cell cycle arrest and apoptosis and preventing the accumulation of damages induced by genotoxic agents and oxidative stress [102–104]. Therefore, the loss of FOXO plays a fundamental role in the progression of cancer. FOXO also acts as a critical modulator of metastasis and angiogenesis, two factors that are indispensable for cancer progression and establishment. The relationship between low FOXO expression and increased cancer metastasis was also demonstrated [105, 106]. FOXO1 and FOXO3a are the predominant FOXO factors in endothelial cells, inhibiting endothelial tube formation and migration [107] (Fig. 2).
2. Promising treatments in the PERK pathway

Firstly, the PERK-eIF2α-ATF4 signaling pathway is responsible for cancer growth and resistance against curative treatment. The PERK-eIF2α-ATF4 UPR branch also increases tolerance in cancer cells to hypoxic stress. Also, the PERK-eIF2α-ATF4 signaling pathway in cancer cells mediates the up-regulation of VEGF-A transcription [108]. Therefore, genetic and pharmacological manipulation of the PERK-eIF2α-ATF4 signaling pathway could be designed for CRC therapy. Next, the suppression of PERK-Nrf2-ARE and PERK-Nrf2-Keap1 could be novel strategies for CRC therapy as well. Besides, both PERK and ATF6 can be activated to trigger CHOP [109], which may be a promising strategy for CRC therapy. Under sustained ER stress, hyper-oxidation is triggered in the ER lumen, resulting in H_2_O_2_ leakage into the cytoplasm and ROS induction [110]. Generally, cancer cells produce higher expression of ROS compared to normal cells. Therefore, increasing ROS levels render cancer cells more susceptible to ER stress, contributing to ER stress-mediated apoptosis [111]. Thus, ROS activation could serve as a CRC therapy target that cannot be ignored [112–114].

### ATF6 pathway

A large body of evidence suggests that ATF6 activation has no obvious paradoxical outcomes, primarily induces cytoprotective responses, such as ER biogenesis, chaperone up-regulation, and unfolded/misfolded proteins degradation [38, 115]. Indeed, enhanced the ATF6 fragment’s nuclear translocation, termed ATF6p50, is observed in various cancer types, and the overexpression of ATF6p50 has been validated to be correlated with a higher probability of metastasis and relapse [116, 117].

1. ATF6 may be a discriminator of low-grade dysplasia (LGD) and inflammatory regenerative epithelium in ulcerative colitis (UC) patients.

High levels of ATF6 are associated with reduced time of disease-free survival in patients with CRC. In studies of nATF6IEC mice, the experiment found that sustained activation of ATF6 in the colon promoted dysbiosis and microbiota-dependent tumorigenesis [118]. However, experiments performed with germ-free mice demonstrated that ATF6-activated UPR in the epithelium requires intestinal microorganisms for tumor formation. Though the diagnosis of LGD is critical in the management of UC, it is usually arduous to distinguish LGD from the inflammatory regenerative epithelium. There is increasing evidence that levels of ATF6 are elevated in lesions undergoing a typical pre-cancerous change in the context of both non-UC and UC-associated CRC. Therefore, ATF6 may serve as a promising biomarker to distinguish LGD from inflammatory regenerative epithelium in UC patients [119].

### Anti-CRC drugs through UPR branches

Fucoidan (derived from cladosiphon okamuranus and fucus evanescens) can reduce the viability of HCT116 cells by inhibiting the IRE1-XBP1 pathway and boosting elF2α-CHOP expression [120]. 2-(3,4-dihydroxy phenyl) ethanol (DPE), a phenol antioxidant derived from olive oil, triggers growth arrest and apoptosis in human colon carcinoma HT-29 cells through the activation of the IRE1-JNK pathway and PERK-eIF2α-CHOP pathway [121]. The PERK-ATF4-CHOP UPR branch is proven to be activated by small-molecule inhibitor 42,215. Potent anticancer activity of 42,215 has been well elucidated in human colon adenocarcinoma and CCD 841 CoN regular human colon epithelial cell lines [122]. Brefeldin A (BFA) is an antibiotic known to block protein transport and induce ER stress in eukaryotic cells. BFA can also effectively suppress the progression of colorectal cancer during the tumorigenesis and metastasis stages via the up-regulation of CHOP [123]. Resveratrol (3,4′,5 tri-hydroxystilbene), a naturally occurring polyphenolic compound highly enriched in grapes and red wine, has been revealed to induce anti-proliferation and apoptosis in human cancer cell lines. It may be a desired drug against CRC via activation of eIF2α, in parallel with CHOP cleavage of caspase-4 [124]. Piperine (from piper nigrum Linn and piper longum Linn) generates ROS, CHOP, JNK, cytochrome c in HT-29 cells. Further experiments demonstrate that ER stress-mediated apoptosis by piperine is linked with mitochondrial dysfunction [125]. Similarly, curcumin facilitates the expression of CHOP, JNK in HT-29 cells. The underlying mechanism is attributed to the release of intracellular Ca^2+^, mitochondrial dysfunction, and DR5, which may lead to curcumin-induced ER stress-mediated apoptosis [126] (Fig. 3).

BRAF^V600E^ mutations are associated with poor survival of CRC. Oncogene BRAF triggers ER stress and activates UPR pathways through MEK/ERK. The study has demonstrated that ranking with wild type cells, BRAF mutation type cells dependents more on GRP78. The proteasomal inhibitor Carfilzomib (CFZ) and the aggresome inhibitor ACY-1215 (Histone deacetylase 6-selective inhibitor) are potential targeted drugs for BRAF mutation type CRC. Treatment of BRAF mutation type CRC with combined CFZ/ACY-1215 resulted in a better outcome compared to the effect of either agent alone. The study also found that combined CFZ/ACY-1215 treatment resulted in significantly increased expression of eIF2α/ATF4/ CHOP and IRE1α/JNK [127] (Fig. 3).

## Conclusions and perspective

This review focuses on the three branches of UPR signaling pathways that can induce cell survival adaptation or cell apoptosis, but the mechanisms have not been fully elucidated. Researchers have found that IRE1 regulation can proceed independently of regulated BIP release. Moreover, rather than providing the switch that activates the UPR, the IRE1 and PERK luminal domain interaction with BIP may serve a subtler role as a buffer for monomers, thereby stabilizing at an appropriate level the concentration of IRE1 monomers available for activation by unfolded protein ligands. Little is known about how ATF6 responds to ER stress up to the present day. Its ER-luminal domain shows no sequence homology to other proteins. ATF6 associates with BIP and BIP release under conditions of ER stress may contribute to its activation. The ATF6 luminal domain also contains intra- and intermolecular disulfide bonds that may monitor the ER environment as redox sensors. Additional experiments are required to provide more molecular detail into the three UPR regulators’ enzymatic activity. Future structural studies would benefit from understanding how the two domains, residing in two separate cellular compartments, communicate with each other in the absence and presence of ER stress. The key regulators and effectors of the three pathways can influence each other. For instance, each regulator and effector's activation in the pathway may affect the others in multiple pathways. In turn, the expression of a downstream gene may be regulated by multiple upstream pathways. However, the detailed mechanism of cross-link in the UPR is not fully clarified.

The underlying mechanisms that switch the cell survival to cell death under ER stress remain mostly unknown. Under ER stress, which is the critical factor for survival or apoptosis and which pathway plays a critical role in the regulation, also needs further investigation. To the best of our knowledge, CHOP acts as a joint point of the three pathways. In the future, in-depth research associated with it should be further carried out to explore what kind of circumstances UPR can convert adaptation to apoptosis. Besides, the duration and intensity of ER stress have different influences on the three UPR branches. Thus a critical question is how the ER stress sensors integrate information about the duration and severity of the stress stimuli to result in survival or death induction.

Numerous novel stress-independent functions of UPR signaling modules are emerging as contributors to cell physiology and disease in the absence of ER stress, which plays a critical role and needs further exploration. The occurrence of cancer may relate to the disorder of stress-independent functions of UPR. Furthermore, various colorectal cancers exhibit highly heterogeneous. The hypothesis that the status of UPR activation reflects the tumor heterogeneity needs to be further verified.

Nowadays, more and more therapeutic medicines targeting the UPR pathways and their related downstream regulators are emerging. Furthermore, diagnostic techniques and prognostic indicators in CRC also benefit from the inspiration brought by UPR. Nevertheless, it is unknown whether the anticancer natural products through UPR branches exert cytotoxicity on normal cells. It is probably that UPR-targeted therapies facilitate the proliferation of dormant tumor cells or drive cancer cells into dormancy, thereby protecting them from chemotherapy. Therefore, it is crucial to employ other adjuvant therapies that act in synergy with natural products to counteract the chemotherapy resistance. Using these united interventions may serve as the most likely path for successful CRC-prevention and treatment. Additionally, promoting the UPR adaptation of normal cells under high-risk factors might contribute to cancer prevention.

Future studies should focus our understanding on how the UPR interacts with other signal transduction pathways, how the different pathways cooperate with UPR to determine cell fate. For instance, the UPR and autophagy are intimately connected under cell stress conditions. However, it is unclear how UPR interact with autophagy to affect cell fate [128, 129].

The mechanism of UPR in cancer has not been fully elucidated. In light of these problems, the UPR displays the great potential to wait for exploration in CRC.

## Abbreviations

4-PBA: 4-Phenylbutyric acid
5-FU: 5-Fluorouracil
ATF4: Activating transcription factor 4
ATF6: Activating transcription factor 6
BBR: Berberine
BCL-2: B-cell lymphoma 2
Bim: Building information modeling
bZiP: Basic leucine zipper
CFZ: Carfilzomib
CHOP: C/EBP homologous protein
CRC: Colorectal cancer
CREB/ATF: CAMP-response element-binding protein/Activating transcription factor
EDEM: ER degradation-enhancing α-mannosidase-like 1
EGCG: (-)-Epigallocatechin gallate
eIF2α: Eukaryotic translation initiation factor 2
EMT: Epithelial-to-mesenchymal transition
ER: Endoplasmic reticulum
ERAD: ER-associated degradation
Erdj4: ER-localized DnaJ 4
ERO1α: ER oxidoreductin-1 alpha
FOXO: Forkhead box O
GADD34: Gene growth arrest and DNA damage-inducible 34
GCN2: General control nonderepressible-2
GRP78: Glucose-regulated protein 78
GRP94: Glucose-regulated protein 94
HRI: Heme-regulated initiation factor 2 alpha kinase
IRE1: Inositol requiring kinase 1
IRS: Immunoreactivity score
ISC: Intestinal stem cell; ISR, integrated stress response
IκB: Inhibitor of kappa B
JNK: C-Jun N-terminal kinase
Keap1: Kelch-like ECH-Associating protein 1
LGD: Low-grade dysplasia
MDSCs: Myeloid-derived suppressor cells
MEK/ERK: Mitogen-activated extracellular signal-regulated kinase/ extracellular regulated protein kinases
MKK4: Mitogen-activated protein kinase kinase; mTI, mung bean trypsin inhibitor
NF-κB: Nuclear transcription factor-κB
NRF2: Nuclear factor E2 related factor 2
L-OHP: Oxaliplatin
P58IPK: Protein kinase inhibitor of 58 kDa
PDI: Protein disulfide isomerase
PERK: Pancreatic ER eIF2α kinase
PIGF: Placental growth factor
PKR: Protein kinase interferon-inducible double-stranded RNA dependent
PP1: Protein phosphatase 1
RIDD: Regulated IRE1-dependent decay of mRNAs
RNase: Ribonuclease
ROS: Reactive oxygen species
S1P: Site1 proteases
S2P: Site2 proteases
TAp73: P73 gene encodes full-length protein isoform
TNFR: Tumor necrosis factor receptor
TRAF2: Tumor necrosis factor receptor-associated factor
TUD-CA: Tauroursodeoxycholic acid
UC: Ulcerative colitis
uORF: Upstream open reading frames
UPR: Unfolded protein response
VEGF: Vascular endothelial growth factor
VEGF-R2: Vascular endothelial growth factor receptor-2
XBP1: X-box-binding protein 1
XBP1s: X-box binding protein1splicing
